# Black Hydroxylated Titanium Dioxide Prepared via Ultrasonication with Enhanced Photocatalytic Activity

**DOI:** 10.1038/srep11712

**Published:** 2015-07-02

**Authors:** Chenyao Fan, Chao Chen, Jia Wang, Xinxin Fu, Zhimin Ren, Guodong Qian, Zhiyu Wang

**Affiliations:** 1 State Key laboratory of Silicon Materials, Department of Materials Science and Engineering, Zhejiang University, Hangzhou 310027, China

## Abstract

The amorphous TiO_2_ derived from hydroxylation has become an effective approach for the enhancement of photocatalytic activity of TiO_2_ since a kind of special black TiO_2_ was prepared by engineering disordered layers on TiO_2_ nanocrystals via hydrogenation. In this contribution, we prepared totally amorphous TiO_2_ with various degrees of blackness by introducing hydroxyls via ultrasonic irradiation, through which can we remarkably enhance the photocatalytic activity of TiO_2_ with improved light harvesting and narrowed band gap.

Titanium dioxide (TiO_2_), one of the most popular wide-band-gap photocatalysts, which can be used to degrade organic pollutants and produce hydrogen from water under solar irradiation, has attracted widespread attention in the last decades^1^,^2^ Although TiO_2_ has been applied widely in photocatalysis and solar cell due to its excellent optical properties, effective electron transport and photoreaction activity^3^ The large band gap that ranges from 3.2 to 3.7 eV^4^ was a serious limitation of the applications of TiO_2_ for it only makes TiO_2_ effective under UV light, which accounts for less than 5% of the total solar irradiation. There have been several approaches trying to solve this problem. For example, introducing suitable heteroatoms has been proved to be an effective method to narrow the band-gap, which has been actively pursued to vary the chemical composition of TiO_2_ by adding controlled metal^5^,^6^ or nonmetal (such as N^7^, C^8^, F^9^, S^10^) impurities that generate donor or acceptor states in the band-gap. On the other hand, Chen *et al.* reported their work about engineering the disorder of nanophase TiO_2_ derived from hydroxylation by hydrogenation treatment, which created disorder layers on TiO_2_ nanoparticle (NP) surfaces, accompanying with a dramatic color change from white to black and a substantial enhancement of solar-driven photocatalytic activity^11^ Since this pioneering work been published, the hydrogenation treatment on TiO_2_ NPs to create black appearance and narrowed band-gap has triggered an explosion of interests. Some following studies have tried to explore the reasons for the color change and enhanced photocatalytic activity of hydrogenated TiO_2_. Many groups of researchers proved that hydrogenation treatment induced the oxygen vacancies and Ti^3+^ sites in TiO_2_, resulting in the band-gap narrowing and the separation of photo-generated electrons and holes, which remarkably improved the photocatalytic activity of TiO_2_^12^,^13^,^14^,^15^,^16^,^17^ What’s more, Xia *et al.* found Ti^3+^ defects influenced both the photocatalytic activities in methylene blue decomposition and hydrogen generation. Oxygen vacancies benefited photocatalytic methylene blue decomposition, but harmed the photocatalytic hydrogen generation. Ti^3+^ defects displayed a more complicated effect^18^ While Wang *et al.* regarded Ti^3+^ as the recombination center of light-excited electrons and holes. They presented a new approach assisted by hydrogen plasma to synthesize black TiO_2_ with a core/shell structure, and the H-doped amorphous shell was proposed to induce the localized surface plasma resonance and black coloration, which reduced the localized Ti^3+^ states and yielded over an order of magnitude improvement in the effectiveness of solar-driven photocatalysis^19^ Moreover, Chen *et al.* further confirmed that Ti^3+^ was not responsible for the visible and infrared absorption of black TiO_2_, the hydrogenation induced disorder phase and yielded electronic structure changes from the alteration of the orbital overlapping in TiO_2_^20^ The lattice disorder in black TiO_2_ originated from the hydrogenation helping to break up Ti-O bonds on the surfaces of anatase nanocrystals by forming Ti-H and O-H bonds, making the hydrogenated TiO_2_ crystals shrink compared to the original white TiO_2_ crystals^21^ The highly localized nature of the mid-gap states resulted in spatial separation of photo-excited electrons and holes in black TiO_2_, and that accounted for its high photocatalytic efficiency^22^ They also admitted disorder and amorphous phases are apparently still among the most challenging tasks to tackle both experimentally and theoretically. Further investigation may be needed on the underlying reaction mechanisms as well as the physiochemical properties and thus open new applications for amorphous TiO_2_ nanomaterials^23^.

Ultrasonication is an unique technology for generating NPs with attractive properties^24^,^25^,^26^ The chemical effects of ultrasound derive primarily from the hot spots formed during acoustic cavitation: the rapid formation, growth, and the collapse of bubbles in liquid^27^,^28^ This process serves to concentrate dramatically the low energy density of a sound field, Suslick *et al.* have established that the effective temperature reached during bubble collapse was ~5200 K, with a calculated hot-spot lifetime of <2μs^29^ These extreme conditions (local temperature >5000 K, pressure >20 MPa, very high cooling rates >1010 Ks^−1^) conferred sonicated solutions very special properties^30^,^31^,^32^,^33^ It is a simple and energy efficient process for ultrasonication with fast quenching rate and operating at ambient conditions, which has been widely used in chemical^28^,^32^ and biological^33^,^34^ fields to create new materials with improved properties.

In our previous work, we prepared hydroxylated anatase derived from amorphous hydrate, and we found a way to control the degree of disorder of hydroxylated anatase by heating treatment, which enhanced the photocatalytic activity of TiO_2_ because of the disorder that induced by hydrxylation^35^ But it was difficult to obtain pure amorphous TiO_2_ by heating treatment for the easy crystallization. In this contribution, we still took amorphous hydrate that synthesized from Ti(SO_4_)_2_ and ammonia water by simple one-step aqueous reaction as the precursor, and ultrasonication technology was employed to modify the original TiO_2_, which prepared amorphous hydroxylated TiO_2_ with black appearance, large surface area and enhanced photocatalytic activity. Compared to heteroatoms-doping and hydrogenation, ultrasonication avoided their multiple steps, harsh synthesis conditions, or expensive facilities^36^ The pivotal role of ultrasonic irradiation was studied by varying the time of ultrasonication.

## Results and discussion

**Figure 1:**
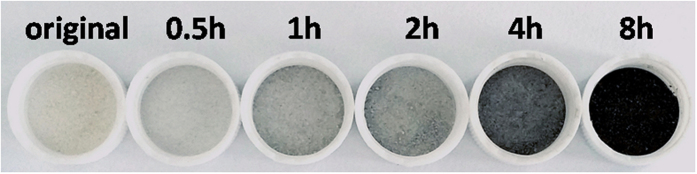
A photo comparing the appearance of original TiO_2_ and ultrasonic treated TiO_2_ for different hours.

The original TiO_2_ prepared by traditional wet chemistry synthesis appeared to be white powder after drying at 80 °C. If the sol of original TiO_2_ was treated under ultrasonic irradiation for several hours, the powder of ultrasonic treated TiO_2_ would turn to black after drying at 80 °C, and the blackness of ultrasonic treated TiO_2_ would be deeper with the extension of ultrasonic time. Figure. 1 and Figure S1 display the appearance of ultrasonic treated TiO_2_ with various degrees of blackness comparing with the original white TiO_2_. It has to be noticed that the power density of employed ultrasonic irradiation was as high as 1500 W/100 mL, and a low ultrasonic power density could not make such changes in color. For example, the ultrasonic washing during the synthesis (see details in the Methods section) could not turn white TiO_2_ to black.

**Figure 2:**
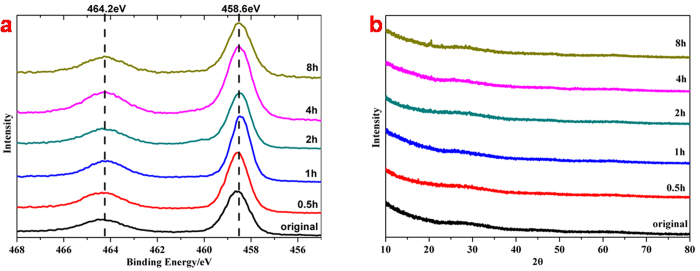
Ti 2p XPS spectra **(a)** and XRD patterns **(b)** of original TiO_2_ and ultrasonic treated TiO_2_ for different hours.

To study the reason for the black color of ultrasonic treated TiO_2_ and their properties, we first compared the X-ray diffraction (XRD) patterns of each sample (Fig. 2b). However, the XRD patterns of samples before and after ultrasonic treatment were quite identical, the particle size hardly grew up and the crystal phase of each sample remained amorphous with the extension of ultrasonic time. Considering the synthesis process, all the samples might be amorphous hydrate with different moisture contents.

The shapes of Ti 2p XPS spectra evidence no significant differences for each sample (Fig. 2a). And the symmetric Ti 2p_3/2_ peaks at 458.6 eV and the Ti 2p_1/2_ peaks at 464.2 eV are attributed to the Ti^4+^ of Ti-O bonds^37^, which demonstrate that Ti atoms had a similar bonding environment before and after ultrasonic treatment, and there was no signs of peak shifting or shoulder^15^,^38^ that assigned to Ti^3+^ exist. So we can actually see the samples of hydrate as amorphous TiO_2_. It was found out that ultrasonic irradiation could accelerate the hydrolysis of TiO_2_ and reduce its crystalline sizes^24^ This is due to the fact that ultrasonic irradiation generated many localized hot spots in the solution and within the sol, which further caused the homogeneous formation of seed nuclei and leaded to a smaller particle size. It was hardly to crystallize for the amorphous TiO_2_ under ultrasonic irradiation of high power density. The Transmission Electron Microscopy (TEM) photographs in Figure S2 prove the totally disorder structure of amorphous TiO_2_ both before and after ultrasonic treatment. Chen *et al.* engineered disorder shells on TiO_2_ NPs to form a core/shell structure by hydrogenation^11^, and our previous work used a contrary pathway to get a similar structure by heating^35^ Here we synthesized by ultrasonication was pure amorphous TiO_2_ as the “shell part” in above works, which was in order to discover the properties of amorphous TiO_2_ without the effects of crystalline structures.

**Figure 3:**
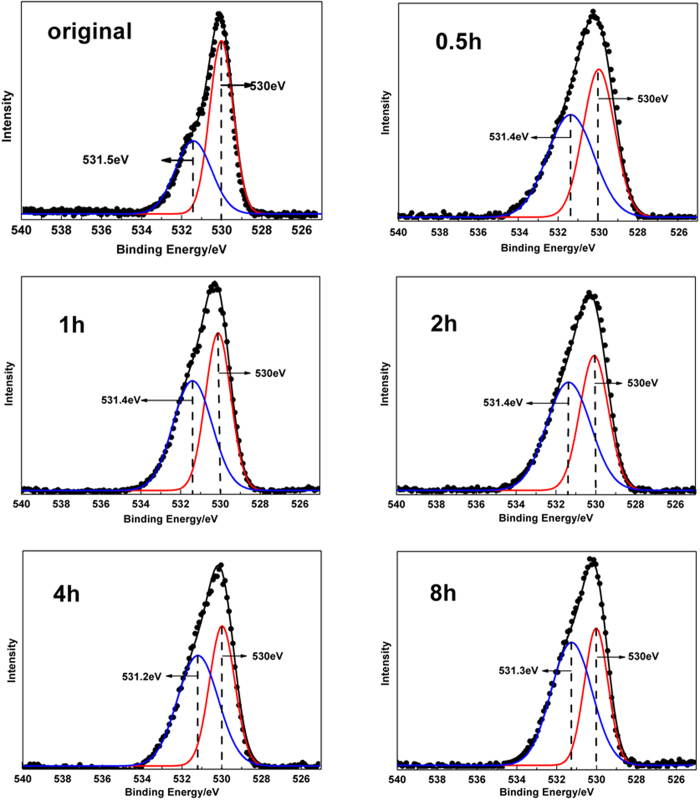
O 1s XPS spectra of original TiO_2_ and amorphous hydroxylated TiO_2_ prepared through ultrasonication for different hours.

As our previous work noticed that this kind of amorphous TiO_2_ was probably induced by the high degree of hydroxylation, we used X-ray photoelectron spectroscopy (XPS) to confirm that conjecture in the present work. Figure S3 shows that before and after ultrasonic treatment, samples of the amorphous TiO_2_ contain the same kinds of elements, which indicates the blackness of ultrasonic treated TiO_2_ was not induced by doped heteroatoms. The O 1 s XPS spectra in Fig. 3 demonstrate similar shapes of original TiO_2_ and ultrasonic treated TiO_2_ for different hours, and the single O 1 s peak in each spectrum can be divided into two symmetric peaks: the one locates at 530 eV is typical for the oxygen of Ti-O bonds in TiO_2_, the other one that locates between 530.9 eV and 532 eV is assigned to the oxygen of Ti-OH bonds^39^ These XRD and XPS (Ti 2p, O 1s) results prove that the samples of hydrate were amorphous hydroxylated TiO_2_. As the area of Gauss peaks in each O 1 s XPS spectrum represents the amount of Ti-O and Ti-OH bonds in amorphous hydroxylated TiO_2_ respectively, we calculated the ratio of Ti-OH/Ti-O bonds and display the results in the second column of Table 1. It is obviously drawn from the results that the degree of hydroxylation of amorphous TiO_2_ became larger with the extension of ultrasonic time, which confirms that the ultrasonication introduced hydroxyls on TiO_2_ and the blackness of amorphous hydroxylated TiO_2_ had a direct relationship with the hydroxylation and amorphism that caused by ultrasonication. Based on the results of XRD and O 1 s XPS, we should assume the accurate molecular formula of the amorphous hydroxylated TiO_2_ as TiO_2-x_(OH)_2x_, in which the “x” represented the degree of hydroxylation of each amorphous sample. Combined with the relationships that a TiO_2_ molecule contains two Ti-O bonds averagely and a H_2_O molecule was transformed by two Ti-OH bonds, it can be easily calculated that the value of “x” has the same increasing trend as the ratio of Ti-OH/Ti-O bonds in each sample with the extension of ultrasonic time.

In order to prove that the change in blackness of amorphous hydroxylated TiO_2_ was induced by hydroxyls but not N-doping, we used NaOH to replace ammonia water during the process of synthesis as contrast. It could be found out that the appearance of amorphous hydroxylated TiO_2_ synthesized from NaOH was still black after ultrasonication. And if we put the samples of amorphous hydroxylated TiO_2_ prepared through ultrasonication in a muffle to heat at a seriers of temperatures, it would be found out that the color of TiO_2_ turned to white gradually (Figure S4a) and the degree of crystallization of TiO_2_ was enhanced with the heating temperature increasing (Figure S4b). For the amorphous hydroxylated TiO_2_ became white and highly crystalline anatase after heating at 800 °C, if we kept each sample at 800 °C until constant weight, all the Ti-OH bonds in the sample could seem to be transformed to Ti-O bonds completely by dehydration and the weight lost during heating treatment could be used to calculate the value of “x” in TiO_2-x_(OH)_2x_, as well as the ratio of Ti-OH/Ti-O of each sample (see details in Methods section). The calculated results of heating treatment are displayed in Table 2, which matched well with the values of Ti-OH/Ti-O that drawn from O 1 s XPS spectra. The results of heating treatment further confirmed the hydroxyls introduced by ultrasonication were the reason for the blackness and amorphism of TiO_2_.

**Figure 4:**
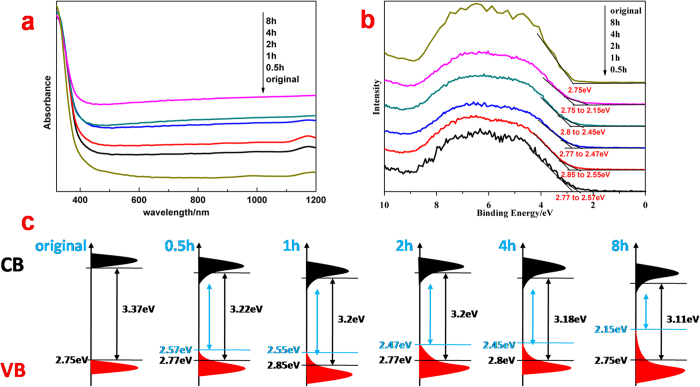
(**a)** UV-Vis absorbance spectroscopy of original TiO_2_ and amorphous hydroxylated TiO_2_ prepared through ultrasonicstion for different hours. **(b)**. VB XPS spectra of original TiO_2_ and amorphous hydroxylated TiO_2_ prepared through ultrasonicstion for different hours. The thin black lines indicate the locations of valance band maximum (VBM) of each sample. **(c)**. The schematic illustrations of DOS of original TiO_2_ and amorphous hydroxylated TiO_2_ prepared through ultrasonicstion for different hours. The black and blue arrows indicate the band gaps before and after localized band bending respectively.

Figure. 4a exhibits the ultraviolet-visible (UV-Vis) absorbance spectroscopy of original TiO_2_ and amorphous hydroxylated TiO_2_ prepared through ultrasonication for different hours. And the amorphous hydroxylated TiO_2_ prepared through ultrasonication for a longer time shows higher absorbance intensity through the whole visible light and near-infrared regions, which explains its deeper blackness in appearance. As the value of band-gap of TiO_2_ was calculated through the equation: *αhν* = A(*hν* − Eg)^*p*^ based on report^40^, where *α* is the absorption coefficient, *hν* is the photon energy, Eg is the optical band-gap, *p* is assumed to be 0.5 for the direct transition and A is a constant concerning the transition probability. We measured the locations of absorption edge of each sample of amorphous hydroxylated TiO_2_, which were transformed into the values of their band-gap and displayed in the third column of Table 1. The density of states (DOS) of amorphous hydroxylated TiO_2_, which describes the number of states per interval of energy at each energy level that are available to be occupied in solid-state and condensed matter physics, were constructed through the results of spectral absorbance (Fig. 4a) and valance band (VB) XPS spectra (Fig. 4b), which are shown in Fig. 4c. The locations of VB edge of each sample that caused by main absorption onset were all around 2.8 eV below the Fermi energy,^17^,^36^ which made the values of intrinsic band-gap (marked by black arrows in Fig. 4c) of each sample only have slightly decrease. Nevertheless, the improved optical absorption of ultrasonic treated TiO2 indicated the localized band bending in DOS, which demonstrated that TiO_2_ under ultrasonic irradiation for longer hours induced larger blue-shift of valance band maximum (VBM) toward the Fermi energy and further resulted in the narrower modified band-gap (marked by blue arrows in Fig. 4c). The similar changes in DOS have occurred in the reported hydrogenated TiO_2_^11^,^17^ As the VB is mainly composed O 2p states, and the conduction band (CB) is mainly formed by Ti 3d states^41^ the long wavelength absorption was attributed to the mid-gap levels from the overlap of O 2p and Ti 3d orbitals^20^,^21^ And the electronic structure changes are the reasons for the blue-shift of VBM toward the vacuum level^11^,^22^, as well as an already predicted CB tail states arising from disorder^11^,^17^. The disorder structure in our amorphous hydroxylated TiO_2_ also yielded electronic structure changes from the alteration of the orbital overlapping, which induced the band tails with the narrowed band-gap. The easier electronic transitions from tailed VB to CB substantially enhanced the optical absorption of amorphous hydroxylated TiO_2_ and finally make the deeper blackness in appearance. This unique structure probably resulted in spatial separation of photo-excited electrons and holes and would significantly enhance the photocatalytic activity^20^,^22^.

It has been proved that structures can play an important role in light harvesting behaviors. The ability of optical absorption depends strongly on the specific surface area. The larger of the surface area, the stronger of the optical absorption^42^. This conclusion matched well with our BET results (4^th^ column of Table 1). The BET measurement also showed the types of physisorption isotherms and hysteresis loops of each sample (Figure S5). All the samples of TiO_2_ exhibited the characteristic features of hysteresis loop and the beginning of the almost linear middle section in Type IV isotherm, which are given by many mesoporous industrial absorbents^43^. And Figure S5 also exhibits Type H2 hysteresis loops of all the samples, which probably indicates that the mesoporous structure of amorphous hydroxylated TiO_2_ was attributed to the pores formed among TiO_2_ NPs. The porosity of each sample of amorphous hydroxylated TiO_2_ that estimated from the pore volume using the adsorption branch of the N_2_ isotherm at P/P_0_ = 0.995^18^,^44^ is displayed in the fifth column of Table 1, which shows the same growing trend as surface area with the extension of ultrasonic time. And it can be drawn from Figure S6 that the pore size distribution of all the samples has a center about 4 nm, while with the extension of ultrasonic time, the pore size distribution of amorphous hydroxylated TiO_2_ became more concentrated. Li *et al.* have reported the ultrasonic treatment could easily form Ti-OH groups in water, giving rise to mesoporous TiO_2_ with a high surface area^44^, which supported our results. These mesopores would allow rapid diffusion of reactants and products during photocatalytic reaction and enhance the speed of photocatalysis.

**Figure 5:**
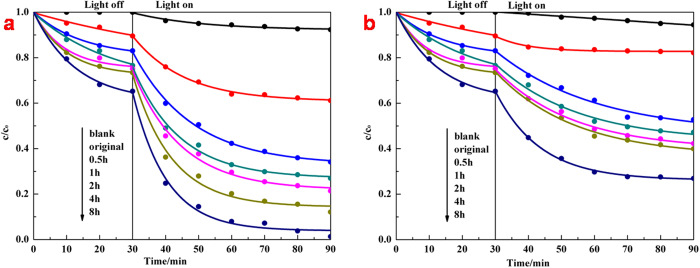
Evaluation of solar-driven **(a)** and visible-light-driven **(b)** photocatalytic activity (AF decomposition) of original TiO_2_ and hydroxylated TiO_2_ prepared through ultrasonication for different hours.

**Figure 6:**
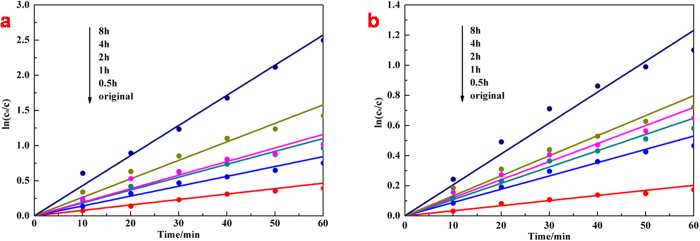
Kinetic plots based on Figure 5a and Figure 5b respectively.

The evaluation of photocatalysis by monitoring the change in optical absorption of acid fuchsin (AF) solution during the process of its decomposing under illumination demonstrated the effects of band-gap narrowing and enhanced optical absorption, surface area and porosity on amorphous hydroxylated TiO_2_ prepared through ultrasonication. We first kept each photocatalytic system under magnetic stirring in dark for 90 min, which found out that the concentration decrease of AF solution caused by physical adsorption of amorphous hydroxylated TiO_2_ mainly occurred at the first 30 min (Figure S7). So we would conduct dark reaction for 30 min before turning light on during photocatalysis measurements. From the photocatalytic results in Fig. 5, it is clear that both the solar-driven and the visible-light-driven photocatalytic activity of TiO_2_ have been improved through ultrasonication. On one hand, the physical adsorption was enhanced because of the growing surface area and porosity. On the other hand, the photocatalytic process is considered as one of the advanced oxidation processes that based on hydroxyl radicals. The degradation of AF solution can be described by an apparent first-order equation with a simplified Langmuir–Hinshelwood model: ln(c_0_/c) = k_a_t^18^, where c_0_ corresponds to the initial concentration of AF solution when light on, and k_a_ is the apparent first-order rate constant. Figure. 6 demonstrate that values of k_a_ of ultrasonic treated TiO_2_ were 1.82, 2.37, 2.5, 3.4, 5.54 times and 2.6, 3.2, 3.54, 3.93, 6.07 times than original TiO_2_ with the extension of ultrasonic time under solar illumination and visible-light illumination respectively. If we eliminated the effects of physical adsorption through calculation without the dark reaction, the pure photocatalytic degradation curves and the corresponding kinetic plots were obtained (Figure S8), which demonstrate the values of k_a_ of ultrasonic treated TiO_2_ in these results were 1.72, 2.29, 2.40, 2.99, 5.78 times and 2.89, 3.79, 4.34, 4.4, 7.22 times than original TiO_2_ with the extension of ultrasonic time under solar illumination and visible-light illumination respectively. Considering the photocatalytic results of two methods and the surface areas of ultrasonic treated TiO_2_ were 1.05, 1.17, 1.29, 1.43, 1.97 times than original TiO_2_ with the extension of ultrasonic time from Table 1, it indicates that the longer of the ultrasonic treatment, the fewer of the effects on photocatalytic activity by physical adsorption. What’s more, the visible-light-driven photocatalytic activity showed a higher degree of enhancement than solar-driven photocatalytic activity of amorphous hydroxylated TiO_2_ after ultrasonic treatment for a same time. The UV illumination on photocatalysts with photons possessing higher energies than the band gap energy could generate electrons and holes in the valance band and surface hydroxyls, which reduced the dissolved oxygen and oxidized organic molecules respectively^45^. Since photoluminescence (PL) emission resulted from the recombination of free charges^12^, we measured the efficiency of photo-generated electrons and holes of each sample through PL spectra (Figure S9). The main PL emission peaks appear at 382 nm and 401 nm with the excitation at 348 nm, and the intensity of peaks is gradually decreased with the extension of ultrasonic time, which indicates enhanced inhibition of the recombination of photo-generated electrons and holes. The amorphous hydroxylated TiO_2_ prepared through ultrasonication effectively reduced the recombination of photo-generated electrons and holes because of the disorder structure acted as the hole traps^22^, which further induced the enhancement of the solar-driven photocatalytic activity. As for the visible-light-driven photocatalysis, the high-energy UV illumination, which generally provided most driving forces in photocatalysis, did not exist, and there was no photo-excited holes generating under visible-light, TiO_2_ was used to help to transmit the charges^46^. The optical absorption improving (Fig. 4a) and the band gap narrowing (Fig. 4c) from the localized band bending of amorphous hydroxylated TiO_2_ prepared through ultrasonication increased the photo-response ranges and light energy harvest, which enhanced the utilization of light and explained the higher degree of enhancement in visible-light-driven photocatalytic activity. Overall, both enhancements in solar-driven and visible-light-driven photocatalytic activity were mainly attributed to the changes in DOS that induced by hydroxylation.

## Conclusions

In summary, we have employed ultrasonic irradiation of high power intensity to prepare amorphous hydroxylated TiO_2_ with various degrees of blackness derived from amorphous hydrate that synthesized through one-step aqueous reaction. With the extension of ultrasonic time, there would be more hydroxyls introduced on amorphous TiO_2_, which changed the electronic structure and further induced the localized band bending with the improvement of optical absorption as well as the band gap narrowing, making the deeper blackness of amorphous hydroxylated TiO_2_, accompanied with the growing surface area and the concentrated pore size distribution. The changes in structure and DOS of amorphous hydroxylated TiO_2_ prepared through ultrasonication could enhance both of the solar-driven and visible-light-driven photocatalytic activity of TiO_2_ effectively.

## Methods

### Preparation of TiO_2_ samples

The precursor of amorphous hydrate was prepared through titanium sulfate (Ti(SO_4_)_2_) and ammonia water reacting in aqueous phase at ice-water bath. Every 100 mL of the Ti(SO_4_)_2_ solution contained 8.0 g solute and the concentration of the ammonia water was 4 mol/L. 12mL of the prepared Ti(SO_4_)_2_ solution and 20 mL of the prepared ammonia water were added into 100 mL of deionized water. Then the system reacted at ice-water bath for 2 h under magnetic stirring to control the synthetic rate. If NaOH was used to replace the ammonia water as contrast, the system would contain 12 mL of the prepared Ti(SO_4_)_2_ solution and 2.0 g of solid NaOH. Other steps and conditions were exactly the same. After the one-step aqueous reaction finishing, the solution was centrifuged (5500 rpm, 8 min) and ultrasonic washed (100 W, 20 min) by deionized water, and the solution after ultrasonic washing could be dried at 80 °C to get the powder of original TiO_2_.

In order to prepare the amorphous hydroxylated TiO_2_ through ultrasonication, the solution after ultrasonic washing would be sent into an XH-300 UL ultrasonic synthesis machine (Xianghu Science and Technology Development Limited Company, Beijing). The ultrasonic process was conducted with an ultrasonic probe and a thermocouple inserting into the solution. During the ultrasonic treatment, the reaction mode was set as constant temperature at 80 °C with an output power density of 1500 W/100 mL, and the duration of ultrasonication could be 0.5 h, 1 h, 2 h, 4 h and 8 h. The solution after ultrasonic treatment was dried at 80 °C to get the powder of amorphous hydroxylated TiO_2_ with various degrees of blackness.

### X-ray diffraction (XRD)

XRD measurement was performed on all the samples of amorphous hydroxylated TiO_2_ using an X’Pert PRO diffractometer operating at 3 kW and a Cu K_α_ radiation source. The scan range was 10°~80° and the step size was 0.02 deg/min.

### X-ray photoelectron spectroscopy (XPS)

All Ti 2p, O 1s and VB XPS spectra were measured by an Escalab 250Xi spectrometer operating at an Al K_α_ radiation source. The binding energy was corrected for specimen charging by referencing the C 1 s peak to 284.6 eV. And the accuracy of the binding energy was 0.02 eV.

### Diffuse reflectance UV-Vis absorbance

The powders of samples were pressed in a round glass model and a BaSO_4_ disk was used as reference material for background measurement. All samples were measured by a Shimazu UV-4100 spectrophotometer, scanned from 300 nm to 1200 nm and the scanning speed was 300 nm/s.

### Heating treatment

Each sample would be dried in a vacuum oven at 110 °C for 24 h to remove the physical water on surface as clearly as possible before the measurement. The weight after drying was noted as the original weight of each sample (W_1_). Then each sample would be sent into a muffle and heated at 800 °C for 3 h to remove the hydroxyls, making the phase totally crystalline, and we weighed each sample again (W_2_) to calculate the value of “x” through the equation: TiO_2-x_(OH)_2x_→TiO_2_ + x[H_2_O]. Considering quantitative relation in dehydration, x = n(TiO_2_)(W_1_-W_2_)/n(H_2_O)W_2_, where n(TiO_2_) and n(H_2_O) represent the molar weight of TiO_2_ and H_2_O. And n(Ti-OH) = 2n(H_2_O), n(Ti-O) = 2n(TiO_2_)-n(Ti-OH), so the ratio of Ti-OH/Ti-O equals to the value of x/(1-x).

### BET surface area and porosity analysis

The surface area and porosity of TiO_2_ was measured by a Trstar II3020 BET and porosity analyzer, and all samples should be preprocessed at 100 °C to clean the surface. The pore volume was calculated using the adsorption branch of the N_2_ isotherm at P/P_0_ = 0.995 to multiply a constant 0.001547. And the porosity equals to the ratio of pore volume/sample volume.

### Photocatalysis

The photocatalytic activity of each sample was measured by monitoring the change in optical adsorption of acid fuchsin (AF) solution during the process of its decomposing under illumination of a xenon lamp (the illumination current was 20A). The original concentration of the AF dyestuff solution was 0.0134 g/L, and each photocatalytic system contained 150 mL of the AF solution and 0.05 g powder of TiO_2_ as photocatalyst. We kept each system under magnetic stirring for 90 min to obtain the adsorption curves, and based on the results, the whole system needed a dark reaction for 30 min and followed by reacting under illumination for 60 min. On the other way, we didn’t conduct the dark reaction before, but eliminated the physical adsorption using dark curves through calculation to obtain pure photocatalytic degradation curves and the corresponding kinetic plots. The illumination of a xenon lamp was used to simulate the solar irradiation, and if we settled a color filter on the light source, the visible-light irradiation could be selected. Every 10 min, the UV-Vis absorbance of AF solution would be measured by a Shimazu UV-4100 spectrophotometer (scanned from 300 nm to 800 nm; scanning speed was 300 nm/s) to figure out the concentration decrease of AF solution.

### Photoluminescence (PL)

The powders of each sample were dissolved in absolute ethanol, forming the solution with a concentration of 0.1 g/100 mL. The solution was dropped into cuvettes and measured on a Hitach F-4600 fluorescence spectrophotometer with absolute ethanol as reference. The excitation wavelength was identified at 348 nm, and the scan speed was 240 nm/min.

## Additional Information

**How to cite this article**: Fan, C. *et al.* Black Hydroxylated Titanium Dioxide Prepared via Ultrasonication with Enhanced Photocatalytic Activity. *Sci. Rep.* **5**, 11712; doi: 10.1038/srep11712 (2015).

## Supplementary Material

Supplementary Information

## Figures and Tables

**Table 1:** Some structural parameters of original TiO_2_ and amorphous hydroxylated TiO_2_ prepared through ultrasonication for different hours.

Sample	TiOH/Ti-Oa)	Eg[eV]	Surface area[m^2^g^−1^]	Pore volume[mlg^−1^]	Porosity[%]^b)^
**original**	0.72	3.37	166.43	0.109	24.8
**0.5** **h**	1.02	3.22	174.62	0.128	28.98
**1** **h**	1.08	3.2	195.37	0.168	38.28
**2** **h**	1.23	3.2	214.00	0.183	42.64
**4** **h**	1.28	3.18	238.11	0.228	53.03
**8** **h**	1.54	3.11	328.55	0.251	58.26

^a)^The ratio of Ti-OH/Ti-O was calculated from the peak areas in O 1 s XPS spectra; ^b)^ The value of porosity was calculated from the ratio of pore volume and sample volume.

**Table 2:** Detail information and testing results of heating treatment.

Sample	W1[g]a)	W2[g]b)	Value of “x”c)	TiOH/Ti-Od)
**original**	0.5171	0.4723	0.422	0.73
**0.5** **h**	0.7077	0.6328	0.526	1.11
**1** **h**	0.7045	0.6283	0.539	1.17
**2** **h**	0.5684	0.5052	0.556	1.25
**4** **h**	0.3836	0.3399	0.571	1.33
**8** **h**	0.3698	0.3248	0.616	1.60

^a)^W_1_ represents the original sample weight before heating.

^b)^W_2_ represents the sample weight after heating at 800 °C for 3 h.

^c)^The value of “x” was calculated through n(TiO_2_)(W_1_-W_2_)/n(H_2_O)W_2_.

^d)^The ratio of Ti-OH/Ti-O equals to x/(1-x).
