# Supplementary material for: Black Hydroxylated Titanium Dioxide Prepared via Ultrasonication with Enhanced Photocatalytic Activity
Source: Sci Rep. 2015 Jul 2;5:11712. doi: 10.1038/srep11712 (PMC4488957; doi:10.1038/srep11712)
Supplement: Supplementary Information [file srep11712-s1.pdf]

Supplementary Information

Black Hydroxylated Titanium Dioxide Prepared via Ultrasonication with Enhanced Photocatalytic Activity

Chenyao Fan, Chao Chen, Jia Wang, Xinxin Fu, Zhimin Ren,

Guodong Qian, Zhiyu Wang*

State Key Laboratory of Silicon Materials, Department of Materials Science and engineering, Zhejiang University, Zheda Road 38, 310027, Hangzhou, China

Figures

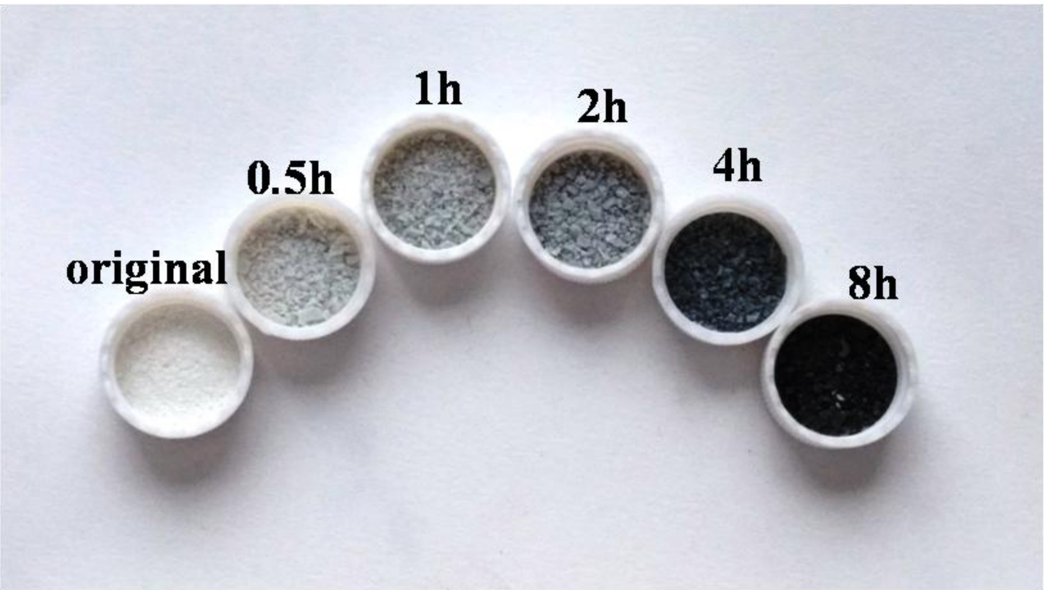

Figure S1. A photo comparing the appearance of original TiO_2 and amorphous hydroxylated TiO_2 prepared through ultrasonication for different hours before grinding.

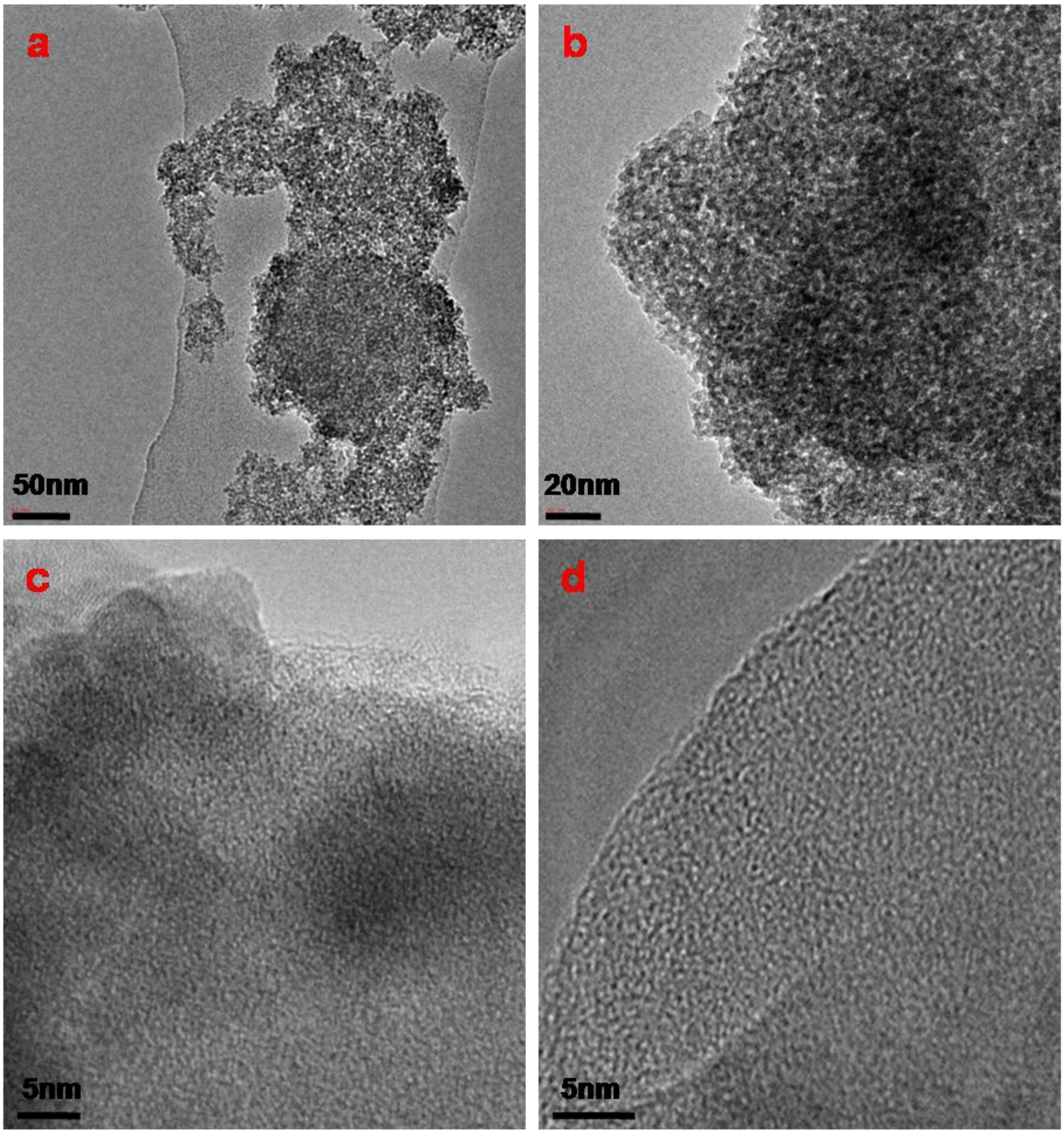

Figure S2. TEM/HRTEM images of original TiO_2 **(a)/(c)** and amorphous hydroxylated TiO_2 prepared through ultrasonication for 8h **(b)/(d)**.

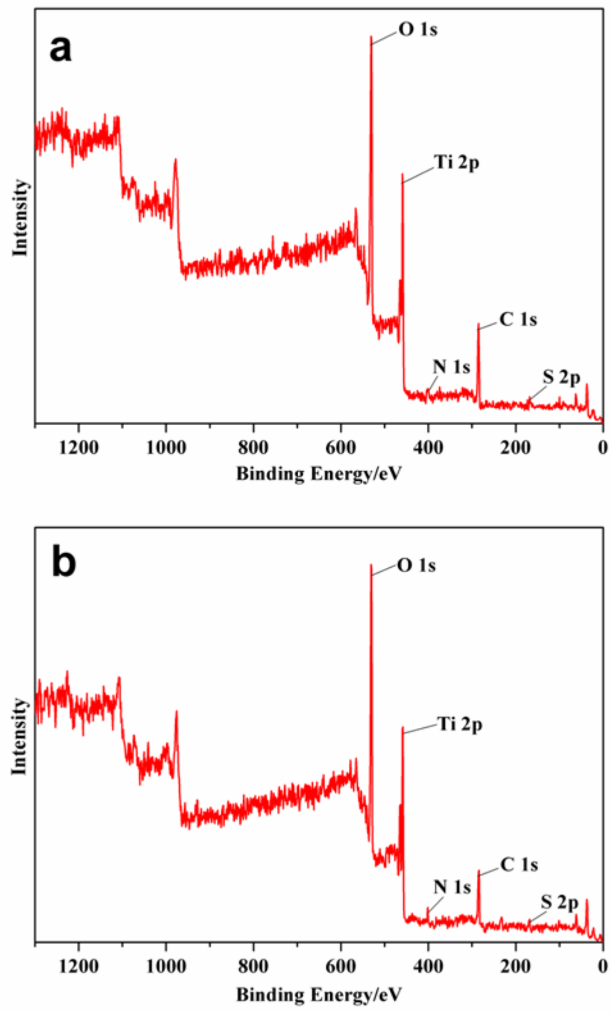

Figure S3. XPS survey spectra of original TiO_2 **(a)** and amorphous hydroxylated TiO_2 prepared through ultrasonication for 8h **(b)**.

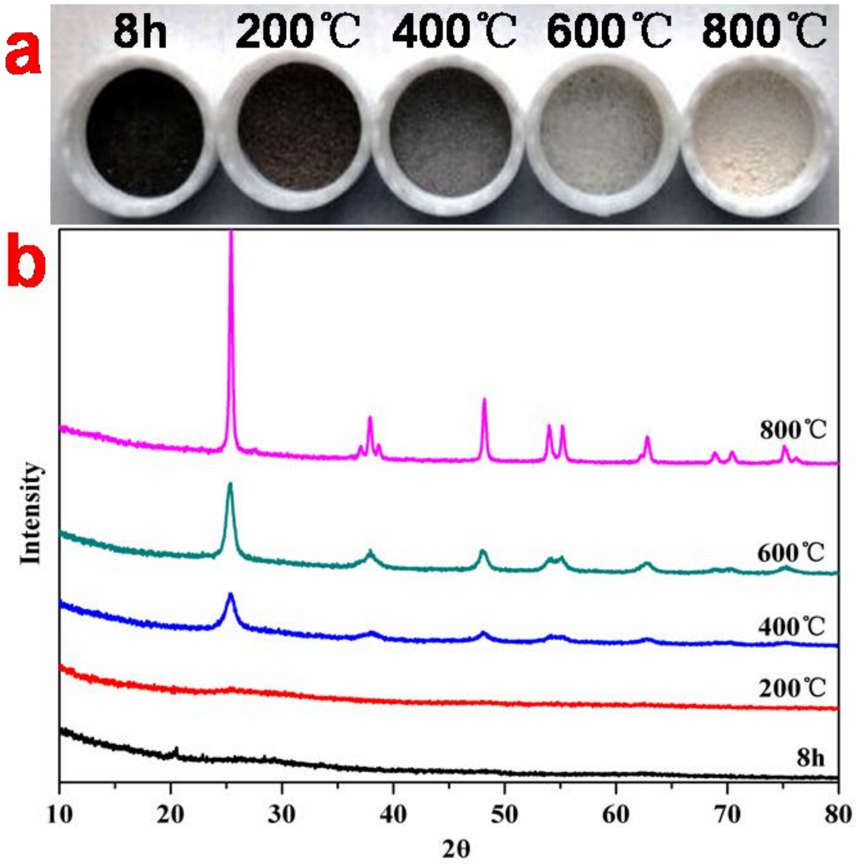

Figure S4. (a) A photo comparing the appearance of amorphous hydroxylated TiO_2 (synthesized from NaOH) prepared through ultrasonication for 8h and it heated at a series of temperatures. **(b)** XRD patterns of amorphous hydroxylated TiO_2 (synthesized from NaOH) prepared through ultrasonication for 8h and it heated at a series of temperatures.

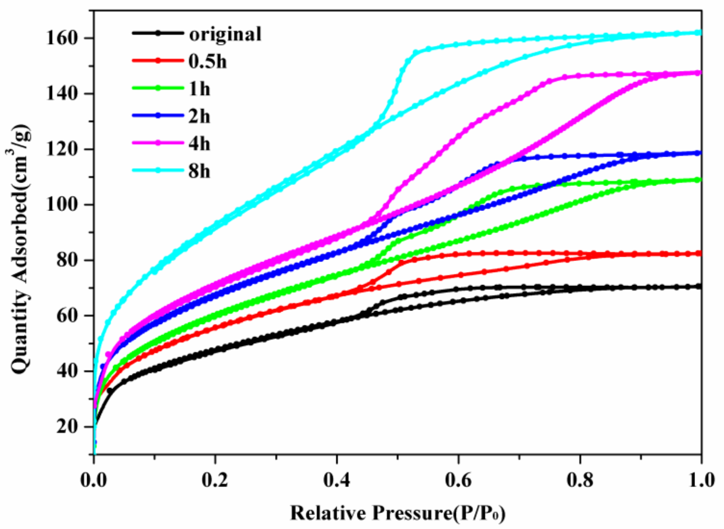

Figure S5. N_2 adsorption/desorption isotherms of original TiO_2 and amorphous hydroxylated TiO_2 prepared through ultrasonication for different hours.

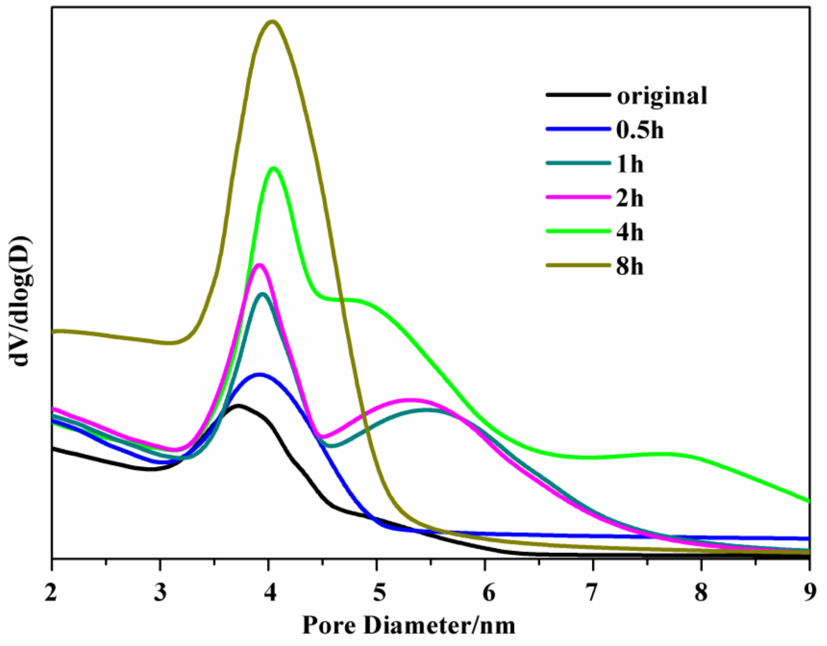

Figure S6. BJH pore size distribution curves of original TiO_2 and amorphous hydroxylated TiO_2 prepared through ultrasonication for different hours that calculated from the desorption branch of the N_2 isotherms.

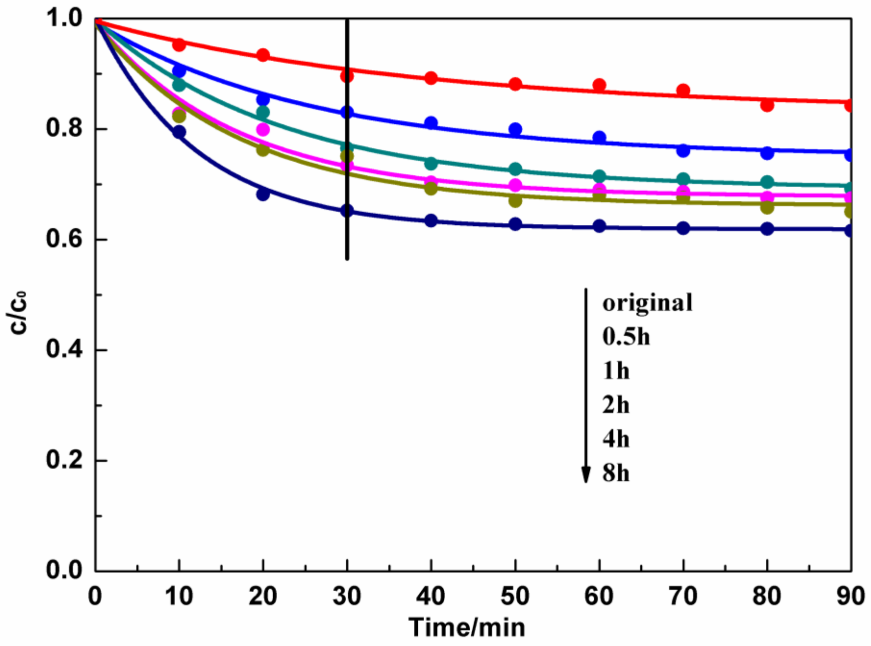

Figure S7. Dark reaction curves (AF adsorption) of original TiO_2 and amorphous hydroxylated TiO_2 prepared through ultrasonication for different hours.

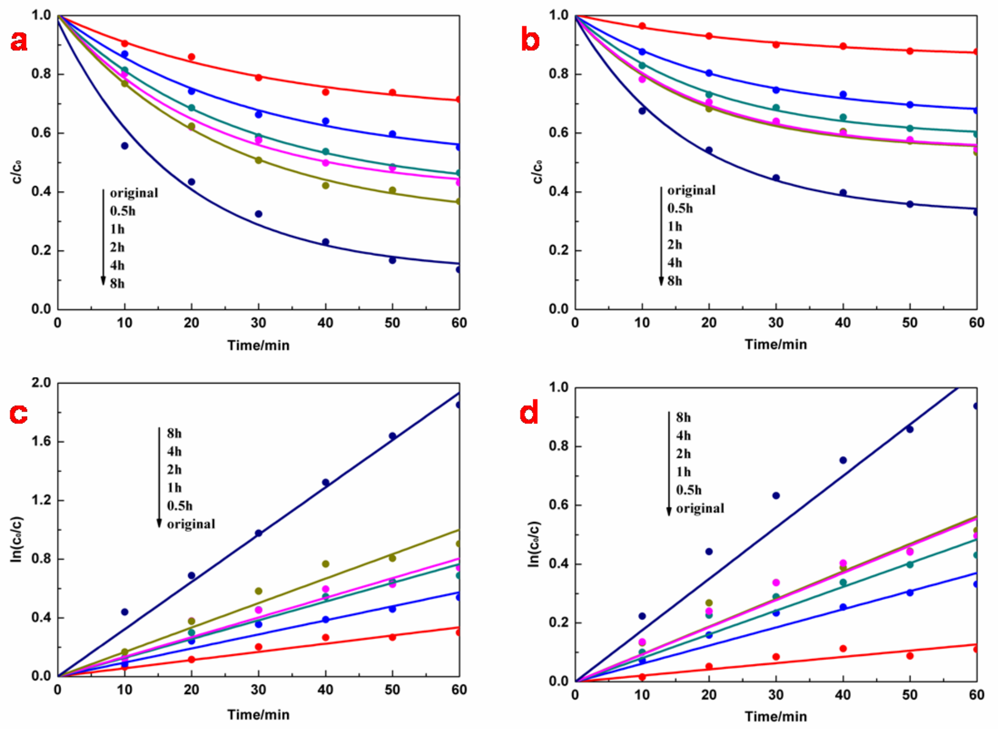

Figure S8. Pure solar-driven/visible-light-driven photocalytic (AF decomposition) degradation curves (a)/(b) and corresponding kinetic plots (c)/(d) of original TiO_2 and amorphous hydroxylated TiO_2 prepared through ultrasonication for different hours.

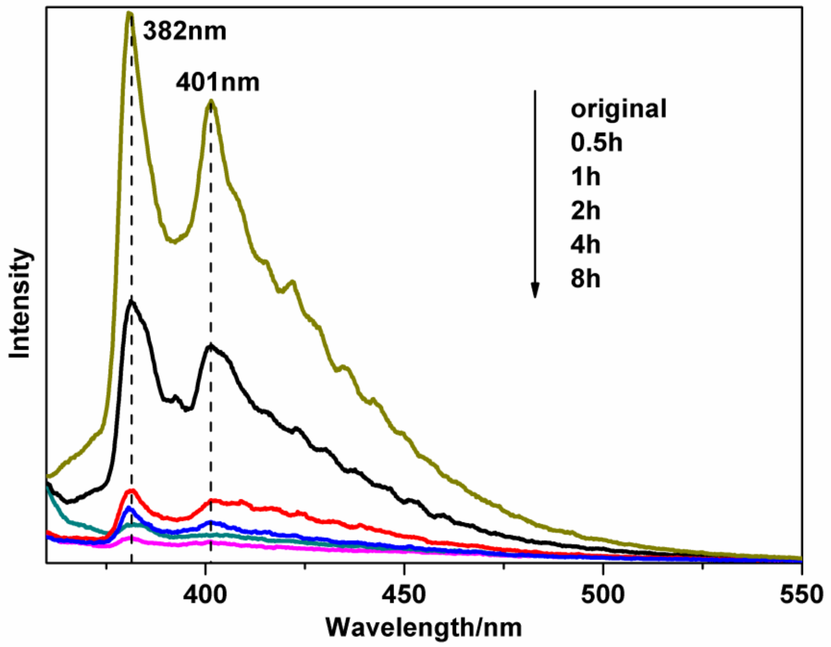

Figure S9. PL spectra of original TiO_2 and amorphous hydroxylated TiO_2 prepared through ultrasonication for different hours.
